# Laparoscopic Management of Double Gall Bladder: A Case Series

**DOI:** 10.7759/cureus.26110

**Published:** 2022-06-20

**Authors:** Mohammad Atif Khan, Kandhala Srikanth, Guru Prasad Painuly, Bhargav Gajula, Jaydeep Jain

**Affiliations:** 1 Surgery, Max Super Speciality Hospital, Dehradun, IND

**Keywords:** symptomatic cholelithiasis, gall stone disease (gsd), laparoscopic cholecystectomy, double gall bladder, duplicate gall bladder

## Abstract

Double gall bladder or duplication of the gall bladder is a rare congenital malformation. It poses a challenge to the surgeon and the radiologist, both in preoperative evaluation and intraoperative management. In the era of minimal invasive surgery, clear knowledge of extrahepatic biliary anatomical variations is very much essential. The operating surgeon should be very careful and overcautious in identifying such variations to prevent untoward biliary tract injury. In this series of two cases, we present the clinical peculiarities, preoperative diagnosis, and laparoscopic management of the duplicate gall bladder.

## Introduction

Duplication of the gall bladder is a rare congenital malformation that results from embryonic dysgenesis of the hepatic antrum which gives rise to the ductal system. The incidence is not exactly known but the available literature denotes a case in every 4000 live births [1]. In the era of advanced laparoscopic and robotic surgical techniques, these congenital malformations predispose to iatrogenic biliary tract injuries if not identified promptly. Established diagnosis preoperatively using magnetic resonance cholangiopancreatography (MRCP) can avoid the intraoperative surprise to the surgeon. The usage of intraoperative cholangiography may help in the detection of malformation and aid in dissection, thereby preventing iatrogenic bile duct injury. Laparoscopic cholecystectomy remains the surgery of choice in management [2]. We present two cases with different clinical and intraoperative dilemmas, medicolegal implications, and laparoscopic management of the duplicate gall bladder.

## Case presentation

Case 1

**Figure 1:**
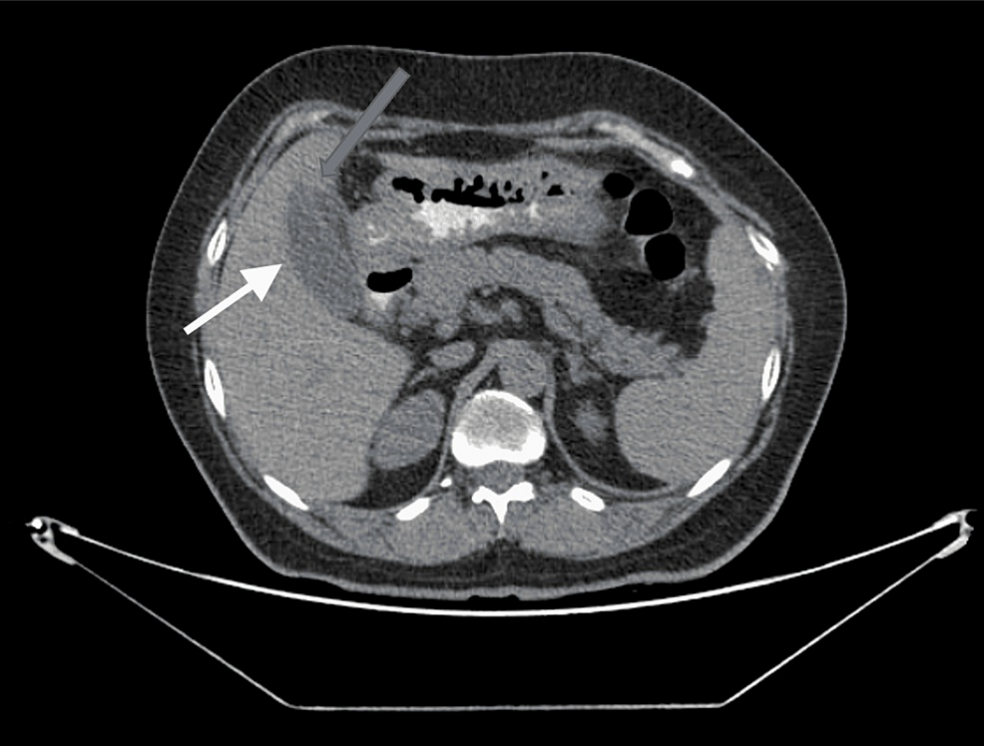
CT abdomen axial view showing the duplicated gall bladder, one of them with the collapsed lumen (grey arrow) and distended gall bladder (white arrow)

A 59-year-old woman from Northern India had been suffering from pain in her right upper abdomen for one year. She had no further concerns and her abdominal examination was unremarkable. Ultrasound of the whole abdomen revealed gall stones and suspected double gall bladder. Computed tomography of the abdomen showed a double gall bladder; one of them was with a collapsed lumen (Figure 1).

**Figure 2:**
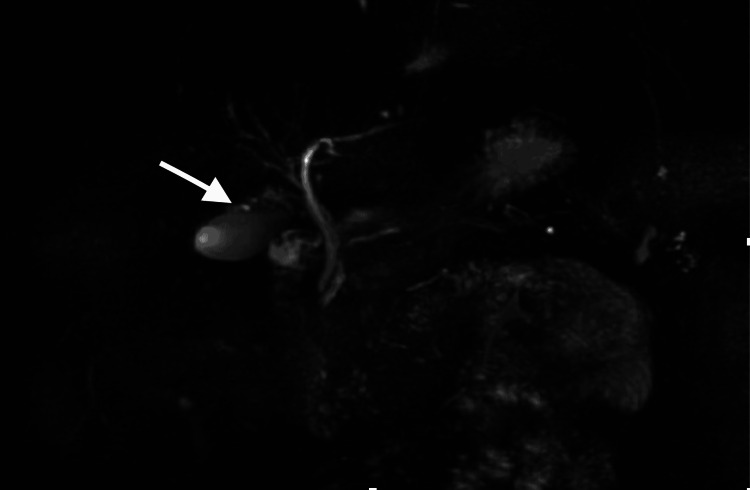
Magnetic resonance cholangiopancreatography (MRCP) coronal view showing the double gall bladder (white arrow) entering the common bile duct

Later, the patient underwent MRCP which showed the duplication of the gall bladder (Figure 2).

**Figure 3:**
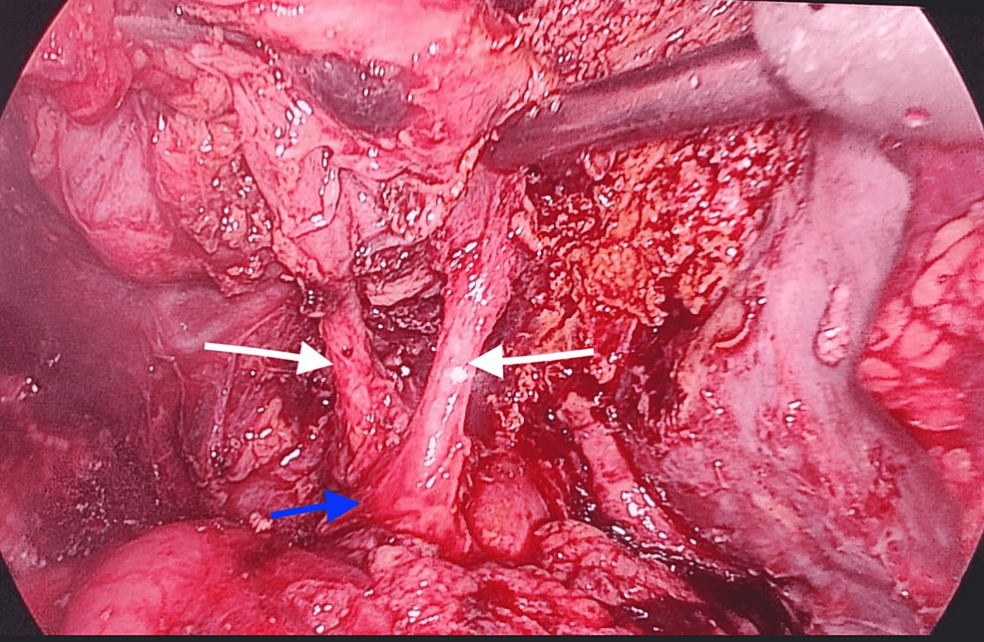
Intraoperative image showing double cystic ducts (white arrows) entering into the common bile duct (blue arrow)

The patient underwent laparoscopic cholecystectomy. Intraoperatively, we found a duplicated gall bladder with two cystic ducts entering the common bile duct (Figure 3).

The postoperative course of the patient was uneventful. On follow-up after two weeks, the patient's recovery was unremarkable. The histopathology was consistent with chronic inflammatory changes in both the gall bladders.

Case 2

**Figure 4:**
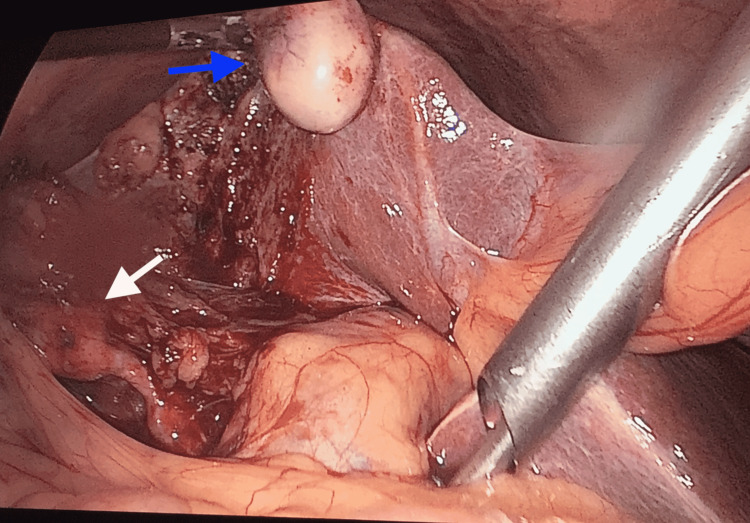
Intraoperative image showing the gall bladder with established hepatocystic triangle (white arrow) and additional accessory gall bladder hanging to the liver's inferior surface (blue arrow)

A 61-year-old female has recurrent abdominal pain in the emergency room of our institute. On clinical examination and ultrasound examination, it proved to be a case of acute cholecystitis. The patient was taken for emergency laparoscopic cholecystectomy. To our surprise, we found an accessory gall bladder-like structure hanging to the liver's inferior surface with a normal gall bladder along with a properly established hepatocystic triangle (Figure 4). Cholecystectomy was done along with the removal of the accessory gall bladder-like structure which had a fibrous band-like connection with the liver and a thin arterial supply.

Porta and supra duodenal areas were explored and no additional structures were found. The postoperative course of the patient was uneventful and the patient was discharged. Histopathological examination of the accessory structure confirmed that of the gall bladder [1].

## Discussion

Duplication of the gallbladder is a rare congenital malformation with an incidence of one in 4000. It is usually detected when it becomes symptomatic, as an incidental finding in laparotomy, autopsy, or imaging studies. Boyden classified gall bladder duplication into three types; Type 1: bilobed gall bladder with incomplete division and one common cystic duct; Type 2: completely duplicated gall bladder with separate cystic ducts that lead to common hepatic duct; Type 3: completely duplicated gallbladder with a common cystic duct entering the common hepatic duct [3].

Being rare, the duplication of the gall bladder is challenging to identify preoperatively and manage intraoperatively. Any disease in duplication gall bladder lacks a specific presentation and is difficult to identify by routine clinical history and clinical examination. The incidence of cholecystitis (acute or chronic), torsion, cholelithiasis, cholecysto-enteric fistula, and malignancy are similar to that of the single gall bladder [4]. Radiologically investigations with routine ultrasound abdomen cannot identify its presence in almost all the cases. This can be attributed to the limitation of the ultrasound technique as it can describe the gallbladder wall and the luminal contents and inadequate visualization of the extrahepatic biliary tree. Moreover, confusion raises among the differential diagnoses like Phrygian cap, gallbladder diverticulum, the fold of gallbladder, Type 2 choledochal cyst, focal collection of pericholecystic fluid, focal adenomyomatosis, intraperitoneal fibrous bands, and dilated cystic duct remnant [5].

Other investigations like MRCP, endoscopic retrograde cholangiopancreatography (ERCP), hepatobiliary iminodiacetic acid (HIDA) scan, and oral cholecystography can detect the double gall bladder preoperatively [6]. MRCP and ERCP are proven to be better but the MRCP has the advantage of a better anatomical description of the biliary tract, the ability to detect any unusual location of the duplicated gall bladder, and is less time-consuming and noninvasive compared to ERCP. MRCP can easily establish Boyden's type of gall bladder duplication [7].

Indications for cholecystectomy in duplicated gall bladder remain the same as in a single bladder. Laparoscopic cholecystectomy is the gold standard approach even though in the initial days, some surgeons preferred an open approach [8]. A safe cholecystectomy strategy with the proper understanding of biliary tract anatomical variation, proper use of energy sources, and bail-out strategies will always decrease the chance of bile duct injury [9]. In doubtful cases, intraoperative cholangiography and laparoscopic ultrasound are to be used for a better understanding of the anatomy intraoperatively [10]. Care should be taken to completely excise the gall bladder with gall stones even though a normal double gall bladder should be excised without fail. This prevents future recurrence, a second surgery, and unwanted medicolegal claims against the surgeons. Cases have been reported of remote presentation with gall bladder stones, acute cholecystitis, and gall bladder perforation in patients who have previously undergone cholecystectomy [11,12]. The postoperative histopathological examination helps in confirmation in cases where duplicated gall bladder is excised from the unusual location at the portal or supra-duodenal areas. The most confusing differential is the Type 2 choledochal cyst, an isolated true diverticulum protruding from the wall of the common bile duct. The only way to differentiate between Type 2 choledochal cyst and double gall bladder is the presence of muscle layer in histopathology examination in the latter entity [3].

## Conclusions

Gall bladder duplication, despite being a rare case, is challenging when presented. Gall bladder duplication should be kept in mind when the routine ultrasound finding is unclear and in this case, MRCP should be done to identify the anatomy. The operating surgeon should be overcautious to appreciate its presence if found intraoperatively and should follow the safe cholecystectomy strategy to prevent bile duct and vascular injury. Both the gallbladders should be excised without fail to prevent recurrent disease. Hence, preoperative identification of a duplicate gall bladder by imaging and knowledge about its differentials helps to avoid surprises during operation. It also prevents a second surgery and unnecessary medicolegal claims against the surgeons.
